# P04.30. Physical inactivity among employees at major academic medical center and university

**DOI:** 10.1186/1472-6882-12-S1-P300

**Published:** 2012-06-12

**Authors:** G Birdee, D Byrne, P McGown, L Rolando, M Holmes, M Yarbrough

**Affiliations:** 1 Vanderbilt University Medical Center, Nashville, USA

## Purpose

In the U.S., one out of three adults are physically inactive (PIA), which is associated with increased mortality. The workplace is a potential venue to promote physical activity.

## Methods

We examined data from the 2010 health risk assessment (HRA) among Vanderbilt University employees who are enrolled in the employer insurance plan. The HRA is a 39-item online questionnaire developed by Wellsource, Inc. In 2010, 80% of eligible employees completed the HRA (n=16,976). We analyzed characteristics of PIA among employees, defined as individuals who reported exercising less than one time a week in the last year. We used bivariable models to examine the associations of PIA with sociodemographics, health status, chronic medical conditions, mental health, and health behaviors.

## Results

Among the 16,976 employees who completed the HRA, 3,002 individuals reported physical activity less than once a week (18%). PIA levels were higher among women than men (OR 1.3 [1.2-1.4]) and older individuals. Non-faculty employees were less active than faculty employees (OR 2.3 [2.0-2.6]). Individuals who were PIA as compared with physically active reported higher prevalence of hypertension (24% versus 15%, respectively), high cholesterol (14% versus 9%, respectively), history of heart disease, cancer, or stroke (12% versus 7%, respectivley), and trouble coping with stress (15% versus 7%, respectively). Higher rates of absenteeism and dissatisfaction with work were reported among PIA than active employees. Individuals who reported fair or poor overall health were five times more likely to be PIA than active (OR 4.8 [4.0-5.6]).

## Conclusion

Nearly one out of five employees were PIA and reported higher stress, chronic medical conditions, absenteeism, and dissatisfaction with work compared to physically active employees. Future longitudinal research is needed to identify barriers to physical activity and if increases in physical activity are associated with improved well-being, decreased incidence of chronic health conditions, and/or reduced health utility costs among employees.

